# Artificial intelligence and glaucoma: a lucid and comprehensive review

**DOI:** 10.3389/fmed.2024.1423813

**Published:** 2024-12-16

**Authors:** Yu Jin, Lina Liang, Jiaxian Li, Kai Xu, Wei Zhou, Yamin Li

**Affiliations:** Department of Eye Function Laboratory, Eye Hospital, China Academy of Chinese Medical Sciences, Beijing, China

**Keywords:** glaucoma, artificial intelligence, screening, diagnosis, optical coherence tomography

## Abstract

Glaucoma is a pathologically irreversible eye illness in the realm of ophthalmic diseases. Because it is difficult to detect concealed and non-obvious progressive changes, clinical diagnosis and treatment of glaucoma is extremely challenging. At the same time, screening and monitoring for glaucoma disease progression are crucial. Artificial intelligence technology has advanced rapidly in all fields, particularly medicine, thanks to ongoing in-depth study and algorithm extension. Simultaneously, research and applications of machine learning and deep learning in the field of glaucoma are fast evolving. Artificial intelligence, with its numerous advantages, will raise the accuracy and efficiency of glaucoma screening and diagnosis to new heights, as well as significantly cut the cost of diagnosis and treatment for the majority of patients. This review summarizes the relevant applications of artificial intelligence in the screening and diagnosis of glaucoma, as well as reflects deeply on the limitations and difficulties of the current application of artificial intelligence in the field of glaucoma, and presents promising prospects and expectations for the application of artificial intelligence in other eye diseases such as glaucoma.

## Introduction

Glaucoma is a disease characterized by optic nerve damage and visual field defect, and it is also the world’s first irreversible blinding eye disease (1–3). The main cause of glaucoma is the development of blockage and obstruction of the circulation process of aqueous humor in the eyeball, which leads to increased intraocular pressure (1). Glaucoma is different from other eye diseases in that it has insidious onset and no obvious clinical manifestations and signs in the early stage (4). Therefore, once glaucoma shows the related symptoms, the eye has suffered irreversible and harmful vision reduction and vision loss, and may even lead to blindness, which seriously has a huge impact on the life and health of the population (5, 6). Glaucoma can also lead to many complications that further aggravate eye vision damage, such as infection, fear of light and so on (7, 8). It has to be said that with the increase of many disadvantageous factors including refraction and bad living habits, the prevalence and risk of glaucoma are also gradually increasing (9). Therefore, the screening, diagnosis and treatment of glaucoma are of great significance to human eye health.

In the past, artificial intelligence (AI) was only defined as a kind of science and engineering that made only machines (10, 11). However, with the development of science and technology, artificial intelligence has gradually become the representative word of machine learning and deep learning (12–14). Due to the continuous innovation of deep learning and machine learning technologies, artificial intelligence has made significant progress and has been applied in ophthalmology, cancer diagnosis, drug synthesis, molecular targeting, genomic medicine, proteomics medicine and other fields, especially in image recognition and image diagnosis, and has achieved mature clinical applications (15–17).

The application of artificial intelligence in the field of ophthalmology is very broad, including the diagnosis and screening of a variety of eye diseases (18). This review summarizes the relevant applications of artificial intelligence in the screening and diagnosis of glaucoma, and provides a reliable basis and theory for further clinicians to better formulate treatment plans for glaucoma. At the same time, we have also deeply reflected on the limitations and difficulties of the current application of artificial intelligence in the field of glaucoma. Finally, we put forward good prospects and expectations for the application of artificial intelligence to other eye diseases such as glaucoma.

### Application of AI in glaucoma screening

**Figure 1:**
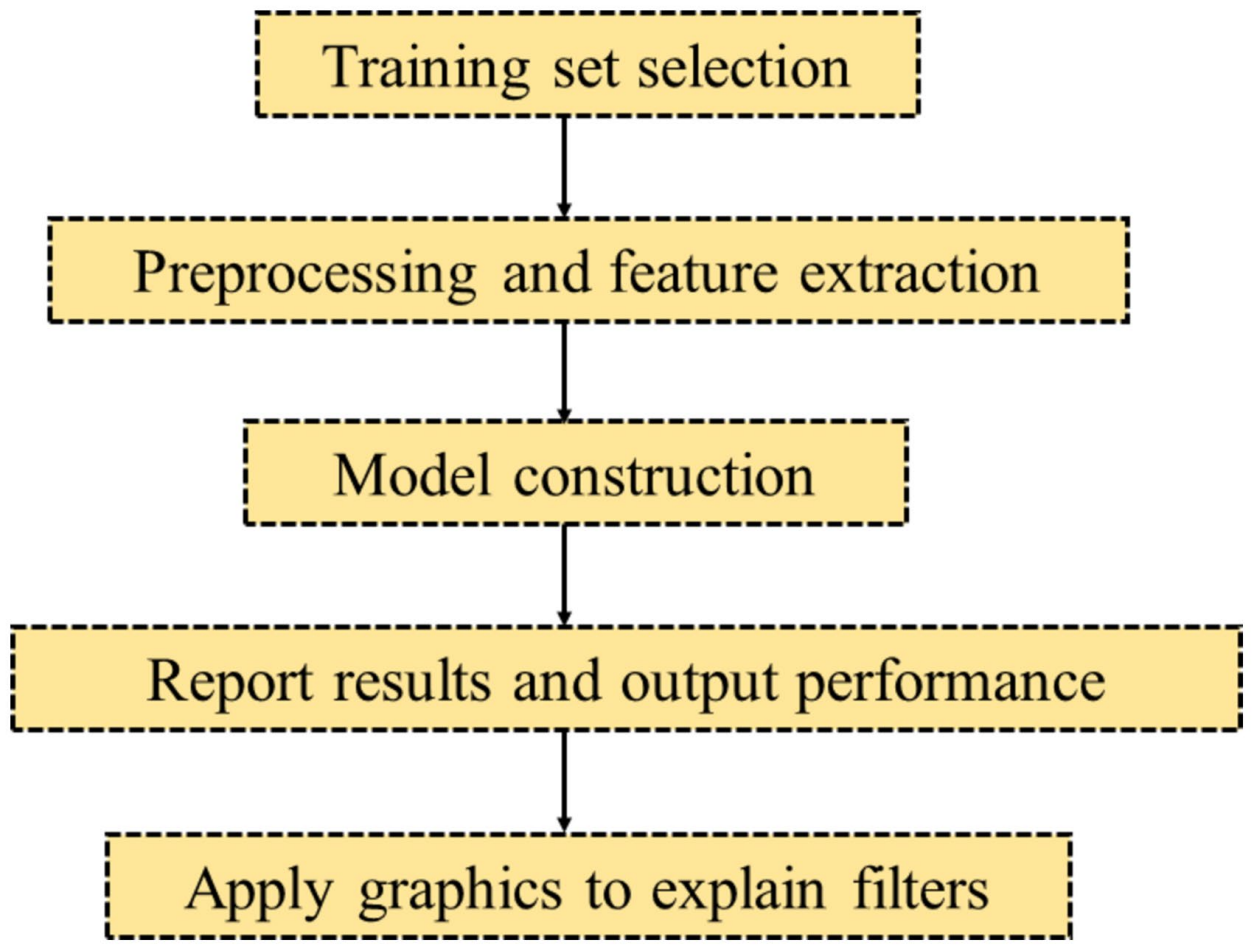
A general procedure of screening for glaucoma.

As the prevalence of glaucoma increases, screening for glaucoma in the population is also critical (19). The application of AI-assisted diagnosis technology to realize early screening of glaucoma can avoid visual impairment of glaucoma patients due to early misdiagnosis or missed diagnosis, and reduce the incidence of glaucoma blindness (20, 21). The screening criteria for glaucoma have always emphasized three principles, which are early detection, early diagnosis and early treatment (22). The traditional screening of glaucoma is a diagnosis by means of tonometer and fundus photography combined with clinical experience (23). This model has the disadvantages of low diagnosis pursuit rate and high screening cost (24). A general procedure of screening for glaucoma in Figure 1. The current AI screening system will provide an efficient and fast algorithm mode, which has a higher detection rate and accuracy rate than the traditional glaucoma screening mode. In addition, the AI screening system can also reduce the cost of examination, greatly easing the financial burden of glaucoma patients.

At present, the main screening techniques for glaucoma include intraocular pressure examination and fundus photography. In the early screening of glaucoma, AI is mainly combined with fundus photography (25). Fundus photography is the most rapid and simple examination method to determine the optic nerve damage in glaucoma (26). Meanwhile, Intraocular pressure (IOP), as the gold standard for glaucoma screening, can also serve as an important auxiliary diagnostic basis for fundus photography (27). In recent years, great progress has been made in the identification of glaucoma by fundus photography. A recent study suggests that improved artificial intelligence could make screening for glaucoma easier by analyzing color fundus photos in a cost-effective way (28). This study proposes a solution for robust glaucoma screening in response to the difficulty of uneven image distribution and low-quality images and their significant degradation of real-world performance (28). At the same time, a study of a large labeled dataset of fundus photo glaucoma screening conducted by AI from the Netherlands also showed that some of the specific features of glaucoma can be recognized and prepared to be captured by AI (29). In the field of glaucoma screening, the development of AI has greatly improved the safety and effectiveness of glaucoma surgery, but AI still needs to continue to overcome shortcomings in terms of targeting, quantification and accuracy. In order to broaden the application of AI in glaucoma or other eye diseases, current researchers and clinicians can make more efforts to improve the targeting accuracy of AI technology.

### Application of AI in glaucoma diagnosis

After screening with AI fundus photography, glaucoma patients need to be referred for further diagnosis (30). Fundus photography is very convenient and economical, which is suitable for assisting large-scale glaucoma screening in areas with backward primary medical system (31). However, further accurate diagnosis still requires the combination of optical coherence tomography (OCT) technology and perimetry results, both of which are objective criteria for determining glaucoma damage (32).

**Figure 2:**
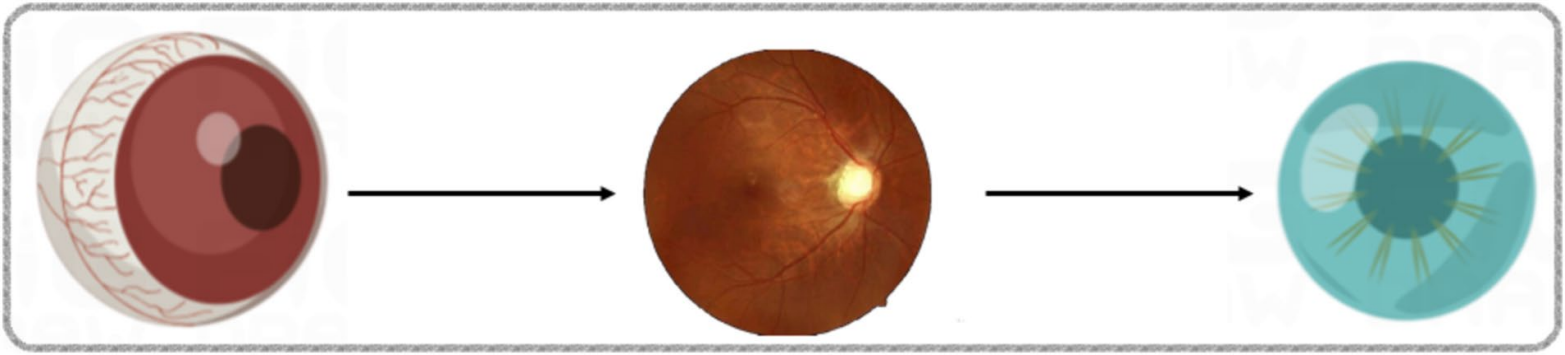
OCT in AI for glaucoma diagnosis.

OCT is a retinal and choroidal imaging technology used to diagnose and monitor eye diseases (33). The principle of OCT is to use interferometry to produce the corresponding real-time correct image in Figure 2. At the same time, this lower power diode laser can correctly hit the emitted infrared light on the tissue to a certain extent, and then return it to the fiber interferometer according to the principle of light reflection (34). By adjusting the time of use and the intensity of the backscattered light, OCT can, in some ways, render a two-dimensional image similar to ultrasound (35). In recent years, the research and application of AI in OCT and perimetry have become more in-depth, which has greatly improved the diagnostic efficiency and accuracy of glaucoma. At the same time, there are also studies that combine the diagnosis of OCT and perimetry to analyze the function and structure, respectively, and propose objective criteria for the diagnosis of glaucoma from the structure and function, so as to obtain more reasonable and accurate diagnosis results. Color fundus image data is enhanced by using histogram equalization (HE) and contrast-limited adaptive HE (CLAHE) image processing. Giancarlo Fortino et al. demonstrated that AI can develop hybrid solutions with image processing and deep learning to ensure the best and most appropriate decisions for glaucoma diagnosis (36). Satish K Panda et al. trained a deep learning network to segment 3 neural tissues and 4 connective tissue layers of the optic papilla of glaucoma, confirming that AI can recognize new biomarkers of the optic papilla of glaucoma for the diagnosis of glaucoma to achieve the diagnosis accuracy of glaucoma (37). The diagnosis of glaucoma by OCT combined with AI still needs a long time to be applied to clinical practice, so it still needs more efforts by the majority of scientific researchers to carry out some interdisciplinary work.

In addition to not only combining with OCT, AI can also be combined with other technologies and detection methods to achieve the purpose of steadily improving the accuracy of glaucoma diagnosis (38, 39). Previous studies have shown that the application of artificial intelligence has a significant effect on the change pattern of the subbasal nerve and corneal aberrations in patients with glaucoma (40). A recent study from Japan shows that artificial intelligence science estimates the time of tear film rupture and effectively diagnoses the occurrence of glaucoma (41). Moreover, artificial intelligence eyeball biomechanical technology can also be used in the diagnose of glaucoma (42). In the future, the research of AI in glaucoma diagnosis needs to be further developed with multi-modal algorithms, because the multi-modal data makes use of more complete inspection parameters and is more comprehensive, and the use of multi-modal data can make a more accurate diagnosis of glaucoma.

### Large language models in the field of glaucoma

The application of large language models (LLM) in the field of glaucoma marks an emerging trend in AI technology in the field of medicine (43). Through deep learning technology, LLM can understand and process a large number of medical text data, such as medical literature, medical records, research reports, etc., thus playing an important role in the diagnosis, treatment and patient management of glaucoma (44). In glaucoma diagnosis, the LLM is able to analyze the patient’s symptoms, history, and test results to provide initial diagnostic recommendations. This helps doctors to judge the condition more accurately, especially in the early identification of high-risk glaucoma patients, the application of LLM has significant value (45). By integrating with electronic medical record system, LLM can automatically extract and analyze medical record data to assist doctors to make more timely diagnosis. In terms of treatment, LLM is able to recommend the appropriate treatment plan for doctors according to the specific situation of patients, combined with the latest medical research results and clinical experience. This not only increases the degree of personalization of treatment plans, but also helps to optimize treatment outcomes and reduce the risk of complications (46). In addition, LLM also plays an important role in the management of glaucoma patients. By building a glaucoma intelligent knowledge base question-and-answer system, LLM is able to provide patients with comprehensive and accurate disease information and self-management recommendations (47). This helps patients to better understand the condition, master self-monitoring and management methods, which can alleviate anxiety to a certain extent and improve the quality of life.

In conclusion, the application of large language models in the field of glaucoma provides a powerful auxiliary tool for doctors, improves the accuracy of diagnosis and the personalized degree of treatment, and provides patients with more convenient and efficient disease management services. With the continuous development and improvement of the technology, the application prospect of LLM in the field of glaucoma will be broader.

### Difficulties and challenges in the application of AI in glaucoma

Currently, AI is being used in the screening and diagnosis of glaucoma (48). To some extent, AI can really improve the screening rate and diagnostic accuracy of glaucoma (49). However, there are still many limitations to the full application of AI in glaucoma. First, the vast majority of hospital-based prospective studies enroll a small number of glaucoma subjects and have limited image integration and segmentation for model development (50, 51). The number of subjects is too small for the AI model to overfit the diagnosis and clinical data of glaucoma patients (52). Therefore, before considering the real clinical application of AI and glaucoma, it is necessary to test on more glaucoma patients to calculate the best model, so that later glaucoma-related research can be carried out more smoothly. Most hospitals have different databases for the combination of AI and glaucoma patients (53).

The number of glaucoma patients in each hospital is limited. Due to the differences in the database including available clinical features, it is difficult to improve the screening rate and diagnostic accuracy of glaucoma by using AI in cross-hospital cooperation (54). Therefore, the field of artificial intelligence for glaucoma urgently needs to develop a universal, publicly available database for further research. If such a database is established, data from glaucoma patients worldwide can be used, reducing the limitations and objectivity associated with a limited number of subjects. In addition, the current acceptance of artificial intelligence for disease management is still limited to a small portion of the population, which is also consistent with the small number of subjects (55, 56). Therefore, in order to mature the application of artificial intelligence in the treatment of glaucoma, promoting the development of artificial intelligence has also become a necessary step and process.

It must be emphasized once again that in the application of artificial intelligence in the diagnosis and treatment of eye diseases such as glaucoma, how to ensure the privacy and security of patient data has also become a hot problem that needs to be solved and solutions need to be found (57, 58). The high cost and low throughput of multimodal diagnostics, as well as the need to consider accuracy and specificity while controlling detection costs, are also issues that will require significant time and effort to address in the future. Finally, in the process of clinical screening and diagnosis of glaucoma, the quality of images obtained will be uneven, which to some extent brings unnecessary difficulties to image processing and integration. Therefore, setting a standard for image output is also conducive to the mature application of artificial intelligence in glaucoma screening and treatment.

### Further advancement and conclusion

The diagnostic ability of AI in glaucoma has been adequately reflected, and the clinical research has gradually become relatively mature. The establishment of an effective AI combined with fundus photography applied in glaucoma screening mode, supplemented by intraocular pressure detection, can greatly improve its screening efficiency and accuracy, and can widely carry out glaucoma screening in various regions (59, 60). In the future, the AI-guided glaucoma screening model will have many advantages, such as high accuracy, high efficiency, high performance and low cost (61). High-quality databases will also be established in various regions, and high-quality databases can simultaneously feed AI to conduct high-quality training tests to improve the performance of AI algorithms and form a benign closed loop (62, 63). In terms of the realization of AI glaucoma screening and AI-assisted diagnosis of glaucoma, the research on single-mode OCT and perimetry has been gradually improved, but the research based on multi-mode data needs to be developed to improve the efficiency and comprehensiveness of AI in diagnosing glaucoma (64, 65). At the same time, the generalization ability of AI needs to be further improved, and the input of invalid data and parameters needs to be reduced (66). In the future, it is necessary to explore a reasonable standard to compare the heterogeneity of different AI in glaucoma, and comprehensively consider gender, age, number of data sets, and concurrent diseases.

AI is also an important part of the prediction of future glaucoma. It has made some progress in the prediction of glaucoma, and will accurately predict the progress of glaucoma patients in the future (67). At the same time, it also has certain application prospects in the humanized treatment of clinical patients. The collection of sufficient longitudinal data can help doctors design personalized treatment plans for glaucoma patients, such as personalized drug courses or surgical recommendations, and control the IOP of patients at safe target IOP through treatment (68, 69). Finally, through the follow-up results adjustment program, the progress of the disease can be controlled, which brings great help to the prognosis of the disease (70). In the future, A will use its ability to detect characteristic patterns in large data sets to select more suitable therapeutic drugs and surgical methods for patients, so as to better humanized intervention and prognosis of glaucoma progression.

To summarize, by combining fundus imaging, OCT, perimetry, and other technologies, AI has demonstrated significant performance in glaucoma early screening, clinical diagnosis, progression prediction, individualized treatment, and prognosis. However, there are certain limits. The era of intelligent diagnosis and treatment is approaching, owing to advances in science and technology, as well as economic strength. It will be popularized in the field of ophthalmology to promote the reduction of the prevalence of glaucoma in the world, bring benefits to patients with glaucoma, and achieve greater social benefits.
